# Pearl millet instant beverage powder enriched with baobab pulp to improve iron and anaemia status of adolescent girls in rural Ghana: a study protocol for a cluster randomised controlled trial

**DOI:** 10.1017/S0007114524001430

**Published:** 2024-09-14

**Authors:** Ambrose Atosona, Christopher Larbie, Charles Apprey, Reginald A. Annan

**Affiliations:** 1 Department of Nutritional Sciences, School of Allied Health Sciences, University for Development Studies, Tamale, Ghana; 2 Department of Biochemistry and Biotechnology, College of Science, Kwame Nkrumah University of Science and Technology, Kumasi, Ghana

**Keywords:** Nutrition, Anaemia, Adolescent girls, Ghana

## Abstract

Iron (Fe) deficiency anaemia is a public health concern among adolescent girls worldwide. Food-to-food fortification may be a sustainable and effective solution to Fe deficiency anaemia. However, the effect of food-to-food fortification on Fe deficiency anaemia reduction is understudied particularly in Ghana. This study seeks to investigate the efficacy of baobab pulp-fortified pearl millet beverage powder in improving the Fe and anaemia status of adolescent girls in Ghana. A three-arm cluster randomised controlled trial design, which will involve 258 anaemic adolescent girls (86/arm) selected through multi-stage cluster sampling in Kumbungu District of Ghana, will be used. Participants in arm 1 will receive 350 ml of baobab pulp-fortified pearl millet beverage, containing 13 mgFe (96 % of average RDA), 18·8 mg vitamin C (30·4 % of average RDA) and 222·1 mg citric acid, while participants in arm 2 will receive similar volume of unfortified pearl millet beverage, once a day, five times a week, for six months. Participants in arm 3 will receive the routine weekly Fe (60 mg)-folate (400 μg) supplementation for six months. Serum ferritin, C-reactive protein and haemoglobin (Hb) of participants will be assessed at baseline and end-line. The primary outcomes will be serum ferritin and Hb concentrations. Secondary outcomes will be prevalence of Fe deficiency, Fe deficiency anaemia and BMI-for-age. One-way ANOVA and paired *t* test will be used to compare means of serum ferritin and Hb levels among and within groups, respectively. This study will provide novel concrete evidence on the efficacy of pearl millet-baobab pulp beverage powder in improving Fe and anaemia status of adolescent girls.

Anaemia among adolescent girls is a global public health concern as about half of them are anaemic^(1)^. Likewise, nearly half of adolescent girls in Sub-Saharan Africa are anaemic^(2)^. In Ghana, the prevalence of anaemia among adolescent girls aged 15–19 years and 10–19 years stands at 48 %^(3)^ and 50·1 %^(4),^ respectively, and as high as 64 % among rural adolescent girls (10–19 years)^(5)^. Anaemia has a deleterious impact on growth and development, work and academic performance, physical and mental capacity, and reproductive health of adolescent girls^(6)^.

Fe deficiency is the main cause of anaemia among adolescent girls^(7)^. Adolescence is a period of fast growth with an acute rise in blood volume, lean body mass and red blood cell mass, which results in a surge in Fe requirement for the synthesis of Hb and myoglobin^(8)^. Adolescent girls are more vulnerable to Fe deficiency compared to their male counterparts because of the monthly loss of Fe through menstruation^(9)^. They are three times more likely to be Fe deficient as compared to adolescent boys^(6)^. Other factors such as poor bioavailability of dietary Fe, low dietary intake of Fe, infectious diseases and parasitic infections also contribute to Fe deficiency^(8,10)^. The determinants of anaemia are context-specific^(11)^, and in Ghana, some of the contextual determinants of anaemia include poor nutritional knowledge, intestinal worms, diarrhoea, food, malaria and poor dietary intake^(12)^. The diets of adolescents, particularly rural adolescents in Ghana, are largely plant-based^(13),^ and bioavailability of Fe from such diets is limited^(14)^; hence, most adolescent girls are unable to meet the increased Fe requirements during adolescence caused by high growth spurt and the loss of Fe each month through menstruation.

In Ghana, Fe food fortification and Fe supplementation are the main interventions in place to address Fe deficiency anaemia among adolescent girls^(15)^. However, these approaches have challenges in terms of acceptability, sustainability and affordability in developing countries^(16–19)^. Food-to-food fortification which utilises locally available foods is recommended as a good alternative measure for addressing micronutrient deficiency in developing countries, as it overcomes the aforementioned challenges of the conventional approaches^(16,17)^. Food-to-food fortification is the addition of a locally available and accessible micronutrient dense food to another locally available and accessible food to enhance the bioavailability of micronutrients to populations^(17)^. In Ghana, food-to-food interventions have been shown to improve Fe status of lactating mothers^(20)^ and anaemia status of children aged 6–9 years^(21)^. However, there is scanty data on food-to-food fortification interventions for improving the Fe status of adolescent girls in Ghana.

Cereals are a major staple in Ghana,^(22)^ and pearl millet is the cereal with the highest amount of Fe (15 mg/100 g)^(23)^; however, only about 7·5 % of the Fe is bioavailable for absorption as a result of anti-nutritive factors and the non-heme nature of the Fe^(24)^. Vitamin C and organic acids such as citric acid can enhance non-heme Fe bioavailability and mitigate the inhibitory effect of anti-nutritive factors (polyphenols and phytates) on Fe absorption^(25)^. The explanations for their effect are dual: (1) they prevent the formation of insoluble and unabsorbable Fe complexes and (2) they reduce ferric (Fe^3+^) to ferrous (Fe^2+^) Fe, which is necessary for the absorption of Fe into mucosal cells^(26,27)^. *In vitro* studies^(28,29)^ have revealed that the addition of baobab pulp to pearl millet-based foods greatly enhances Fe bioaccessibility due to its high vitamin C (200 mg/100 g) and citric acid content (3300 mg/100 g), which is exceptional compared to other fruits and vegetables^(30)^. In the *in vitro* study by Adetola et al.^(28)^, baobab fruit pulp was mixed with pearl millet porridge at a ratio of 15:100, which resulted in a significant increase in the proportion of bioaccessible Fe compared with the unfortified pearl millet porridge. Van Der Merwe et al.^(29)^ also found a similar result in their *in vitro* trial when baobab fruit pulp was mixed with pearl millet flour at a 10:100 ratio and recommended based on their finding that baobab pulp-fortified pearl millet beverages could improve Fe status in women and vulnerable groups, but the extent of improvement can only be determined through clinical trials. However, efficacy trials are yet to be done to confirm these findings as per literature. This study therefore seeks to investigate the efficacy of pearl millet instant beverage powder enriched with baobab fruit pulp in improving Fe and anaemia status of anaemic adolescent girls in rural Ghana. We hypothesise that baobab pulp-fortified pearl millet beverage powder will enhance Fe and Hb status, compared to the unfortified pearl millet beverage powder, in adolescent girls in rural Ghana. We also hypothesise that baobab pulp-fortified pearl millet beverage powder will have a similar or greater effect on Fe and Hb status, compared to the routine Fe -folate supplementation, in adolescent girls in rural Ghana.

## Methods

### Study design

The study will be a three-arm parallel cluster randomised controlled trial. Participants in arm 1 (Intervention group) will receive pearl millet beverage enriched with baobab pulp, while participants in arm 2 (Control group 1) will receive unfortified pearl millet beverage, once a day, five times a week, for six months. Participants in arm 3 (Control group 2) will receive weekly Fe -folate supplementation also for six months. The study outcomes will be assessed twice, at baseline and end-line.

### Study area

The study will be carried out in basic schools in Kumbungu District of Northern Ghana. In Ghana, the rural northern savannah agro-ecological zone has the highest prevalence of anaemia among adolescent girls (64·4 %)^(31)^, with the highest prevalence in the Kumbungu District (75 %)^(32)^.

### Study population

The study population will comprise of adolescent girls aged 10–19 years (pre- and post-menarche girls) attending Upper Primary (Primary 4–6) and Junior High Schools in Kumbungu District. The study is to include both pre- and post-menarche adolescent girls because the average Fe requirement is not greatly different between the two groups (0·3 mg difference)^(7)^. However, data analysis will also be stratified by menarche status to explore any possible menarche status-related differences.

### Inclusion criteria

Adolescent girls aged 10–19 years who have mild or moderate anaemia (8 < Hb < 12 g/dl for girls aged ≥ 12 years and 8 < Hb < 11·5 g/dl for girls aged < 12 years) and consent to participate will be included in the study.

### Exclusion criteria

Adolescent girls who are pregnant/lactating, on anaemia treatment (Fe and folic acid supplements), severely anaemic (< 8 g/dl), severely underweight (BMI-for-age Z-score < −3 sd) and diagnosed with peptic ulcer or sickle cell disease will be excluded from the study. Severe anaemia is a life-threatening condition and requires immediate medical treatment in hospital^(33,34)^, hence the reason to exclude severely anaemic girls from the study. All the excluded severely anaemic girls will be referred to the hospital for treatment. Participants diagnosed with peptic ulcer are to be excluded because the spices (ginger, pepper and clove) in the beverage may irritate peptic ulcers. Additionally, ulcer bleeding might result in anaemia.

### Sampling procedure

**Fig. 1.**
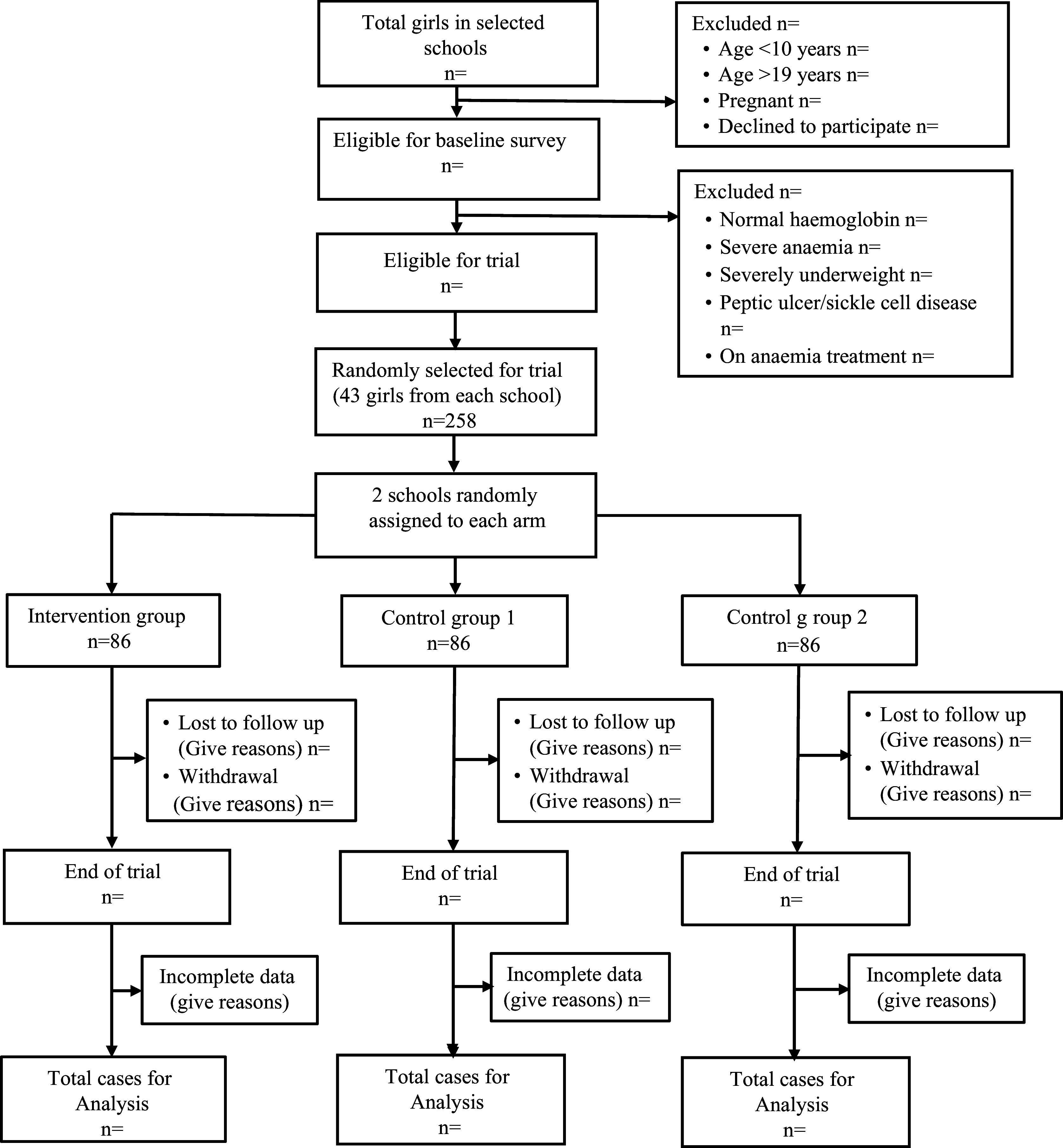
Flow chart of the trial.

A multi-stage cluster sampling technique will be employed in the study. Six communities in the district will be selected through simple random sampling. A school from each of the selected communities will be chosen through simple random sampling, making six schools in all. In the study schools (clusters), all adolescent girls in Upper Primary (Primary 4–6) and Junior High School will be screened based on the inclusion and exclusion criteria to identify eligible participants. Simple random sampling will be used to select the desired number of participants from the eligible participants. The study period is from February to October 2024. Figure 1 depicts the flow chart of the trial.

### Randomisation

An independent statistician will randomly assign the selected schools (clusters) to the 3 arms of study at a ratio of 1:1:1 (2 schools per arm) using the formula =RAND() in Microsoft Office Excel Software. Participants will be informed of their intervention allocation after baseline data collection is completed. This cluster randomisation method will prevent cross-contamination among the study arms through beverage sharing among participants^(35)^. Due to the nature of the intervention, blinding of participants and study team will not be possible; however, the data analyst will be blinded to the allocation of the participants.

### Study timeline

The protocol was developed from October 2022 to February 2023. Initial ethical approval and protocol amendment approval were obtained on 21st April 2023 and 10th January 2024, respectively. The treatment duration (Intervention) of the trial is six months. The specific timelines for participants’ enrolment, interventions, assessments, data analysis and dissemination of results are depicted in Table 1.

**Table 1.** Schedule of enrolment, interventions, assessments, data analysis and results dissemination

	Study period								
	Enrolment	Allocation	Treatment period (6 months)						Close-out
Timepoint (t) (months)	*−t*_*1*_	0	*t*_*1*_	*t*_*2*_	*t*_*3*_	*t*_*4*_	*t*_*5*_	*t*_*6*_	*t*_*7–11*_
Enrolment:									
Eligibility screen	X								
Informed consent	X								
Allocation		X							
Interventions:									
Fortified beverage			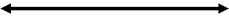						
Unfortified beverage			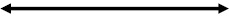						
Fe-folate tablets			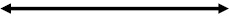						
Assessments:									
Socio-demographics	X								
Dietary intake	X								X
Anthropometry	X								X
Anaemia awareness and prevention	X								
Malaria testing	X					X			
Hb	X								X
Serum ferritin	X								X
C-reactive protein	X								X
Data analysis									X
Results dissemination									X

### Sample size

The formula employed for the sample size estimation is: N = 2S 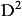 ( 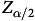 + 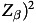 / 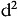 ^(36)^

where N is sample size

sd is standard deviation; estimated to be 20·1 μg/l for serum ferritin^(37)^ and 1·2 g/dl for Hb (anaemia)^(32)^.


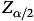
 = 1·96 (From Z table) at a precision of 5 % and CI of 95 %.


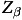
 = 0·84 (From Z table) at 80 % power.

d is effect size; estimated to be 9·4 μg/l for serum ferritin^(38)^ and 0·65 g/dl for Hb (anaemia)^(39)^.

Sample size (Serum ferritin): N = 2 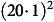 ( 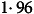 *+* 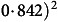 */* 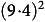 = 72

Sample size (Hb): N = 2 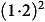 ( 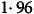 *+* 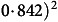 */* 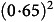 = 53

The larger sample size (N = 72) of the 2 variables (serum ferritin and Hb) will be used for the study.

As a cluster randomised controlled trial, cluster design effect has to be considered in the sample size calculation to improve the power and precision of the study^(40,41)^. The required sample size for a cluster randomisation trial is obtained by multiplying the sample size of the individual randomisation trial (N = 72) by the design effect^(42,43)^. Design effect = 1 + intra-cluster correlation (m-1), where m is the average cluster size. An estimated intra-cluster correlation of 0·0009^(44)^ will be used. The average size of each cluster (school) in the study district is estimated to be 55 adolescent girls, with the number of anaemic adolescent girls in each school estimated to be 43^(32)^, thus m = 43. Design effect = 1 + 0·0009(43–1) = 1·037. Hence, considering the design effect of 1·037 and 15 % attrition rate, the sample size will be 86 participants per arm (258 participants for the 3 arms, with 43 participants to be chosen from each school).

### Preparation and evaluation of composite powder

#### Preparation of pearl millet beverage powder and baobab fruit powder

The pearl millet beverage (unfortified) is a popular Ghanaian beverage. The beverage powder will be prepared using a standardised Ghanaian traditional recipe with minor modifications. The pearl millet (*Pennisetum glaucum*) will be sourced in bulk from the local markets in the study area (Kumbungu District). The millet will be winnowed, debris, sand, and stones sieved out. The millet will be stored in airtight containers at room temperature until use^(45)^. Regarding the beverage powder preparation, the pearl millet (700 g) will be first soaked in clean water (900 ml) for 6 h, drained and rinsed. Ginger (50 g), cloves (4 g) and chilli pepper (4 g) will be added to the soaked millet and ground to flour. The flour will be sun-dried (8 h) and milled to fine powder. For the baobab fruit powder, the fruits will be harvested and crushed to remove the outer shell and the pulp separated from the seeds. The pulp will be ground and sieved to obtain the fine powder. Both the pearl millet beverage powder and baobab fruit powder will be kept in airtight and water-proof containers/bags and stored in a freezer until use^(46)^.

#### Formulation and preparation of beverages

Two powder formulations will be made: (1) baobab pulp-fortified pearl millet beverage powder for the intervention group (arm 1) and (2) unfortified pearl millet beverage powder for the control group 1 (arm 2).

Based on the Ghanaian traditional recipe for the beverage (zomkom), the beverage will be prepared by dissolving the powder mix (60 g millet powder enriched with 9 g baobab fruit powder) in chill/cold water (300 ml) with added sugar (8 g), making a total volume of 350 ml. The amount of baobab powder enrichment was determined based on an acceptability test done among adolescent girls (online Supplementary file 1). The sugar content of the beverage (2·2 g/100 ml) is within the acceptable amount of sugar (≤ 2·5 g/100 ml) in beverages that will have no significant negative impact on BMI and other health outcomes^(47,48)^ and the amount of sugar in the beverage per serving per day (8 g/350 ml) is also far lower than the WHO’s daily recommended maximum amount of free sugar intake for children (50 g, equivalent to 10 % of total energy intake)^(49)^. To test the acceptance of the volume of beverage per serving (350 ml), 40 adolescent girls were fed the beverage over 3 days and all participants comfortably consumed all the 350 ml of the beverage in each feeding session.

The RDA of Fe for adolescent girls aged 10–19 years averages at 13·6 mg (8 mg, 15 mg and 18 mg for girls aged 10–13, 14–18 and 19 years respectively)^(50)^. As in similar studies^(51–53)^, the target of this study is to meet closely the average RDA (13·6 mg) of Fe daily for six months. Hence, the intervention group will receive 350 ml of the baobab pulp-fortified beverage containing 13 mgFe (96 % of average RDA), 18·8 mg of vitamin C (30·4 % of average RDA)^(50)^ and 222·1 mg of citric acid, while the control group 1 will receive similar volume of the unfortified beverage containing 12·4 mgFe (91·2 % of average RDA) once daily, five times a week for six consecutive months. Preparation and formulation of the beverage powders will be done in the Diet Practicum Laboratory at the Department of Nutritional Sciences, University for Development Studies, because of its proximity to the study area.

#### Nutritional content, microbiological quality and shelf life

With regards to the nutrient content of the beverage, proximate (protein, fat and carbohydrate), minerals (Fe, Zn, K, Cu, Mg, Na and P) and organic acids (ascorbic and citric acids) content of the beverage per serving per day (350 ml) were evaluated using Association of Official Analytical Chemists^(54)^, atomic absorption spectrometry^(51)^ and high-performance liquid chromatography^(28)^ methods respectively. Table 2 depicts the nutrient content of the fortified beverage per serving per day (350 ml). Based on microbial quality and shelf life (accelerated technique with moisture content as a determinant) evaluations we performed earlier on the composite powder, the powder is microbiologically safe for consumption (online Supplementary file 2) and has a shelf life of 9 months under room temperature. Also, the composite powder’s vitamin C content showed no significant decline (27·9 mg/100 g to 24·7 mg/100 g, *P* = 0·16) after four months of storage at a temperature of 55°C in plastic bags, indicating its stability.

**Table 2.** Nutrient content of beverage per 350 ml

Nutrient	Amount per serving (350 ml)
Macronutrients (g)	
Fat	7·5
Protein	9·6
Carbohydrate	46·2
Micronutrients (mg)	
Citric Acid	222·1
Vitamin C	18·8
Fe	13
Cu	2
Zn	4·4
Mn	1·5
Na	41·7
Mg	129·8
K	712·9
P	233·9

### Baseline assessment

A pre-tested structured questionnaire will be employed to document information on participants’ socio-demographic characteristics and dietary consumption patterns. Participants’ anthropometric measurements will be taken to assess their nutritional status (online Supplementary file 3). Biochemical indices such as Hb concentration, serum ferritin and C-reactive protein of participants will also be assessed to diagnose Fe deficiency, Fe deficiency anaemia and anaemia. WHO (2020) currently recommends serum ferritin as an adequate marker of Fe status when assessed along with inflammation/infection markers (C-reactive protein and/or *α*-1 acid glycoprotein) and ferritin adjusted as necessary^(55)^.

#### General characteristics

General characteristics of participants such as age, ethnicity, religion, level of education (class), educational and occupational statuses of their parents/guardians, household size, menarche status and age at menarche will be documented.

#### Dietary assessment

A multiple-pass 24-hour dietary recall, which will be repeated in 20 % of a random subsample (for usual intake)^(56,57)^, will be used to collect dietary information from the participants. The 24-hour dietary recall will be conducted on two non-consecutive days, one weekday and one weekend^(58–60)^. Participants will be asked to recall and describe all foods and drinks consumed in the past 24-hours. The amount of food consumed by each participant will be estimated using household handy measures and converted to grams. The total intake of macronutrients and micronutrients including Fe and vitamin C will be quantified using the Ghana Nutrient Analysis Template^(61)^.

Diet quality of participants will also be assessed by means of dietary diversity score^(62)^. Based on the 24-hour recall, the number of food groups each participant ate from will be documented. The 10 food groups recommended by FAO^(62)^ for assessing dietary diversity score of women will be used. Dietary diversity score will be determined by allocating a score of 1 to each food group consumed and a score of 0 to each food group not consumed and a sum total of all scores calculated. Participants who obtain a score ≥ 5 will be deemed to have met the minimum dietary diversity score^(62)^.

Additionally, participants’ dietary pattern will be determined by assessing their frequency of consumption of various food groups over the preceding month, including foods from animal sources, cereals, legumes, fruits, vegetables, sugar-sweetened beverages and snacks. As the participants are teenagers, their mothers will be invited to be present during dietary data collection to ensure accurate data collection.

#### Anthropometry and anaemia awareness and prevention practices

Height (m) and weight (kg) of respondents will be measured and BMI-for-age z-scores computed. Height of participants will be measured without shoes using a stadiometer (Seca, Germany). Weight will be taken in light clothing and without shoes using an electronic scale (Seca, Germany). BMI-for-age z-scores will be calculated using WHO Anthro Plus software and classified as underweight (< −2 sd), normal (–2 sd to +1 sd), overweight (> +1 sd) and obese (> +2 sd)^(63)^. Regarding knowledge and prevention practices related to Fe deficiency anaemia, the FAO questionnaire^(64)^ will be adapted for the study.

#### Haematological and biochemical assessment

At baseline and end-line, about 5 ml of non-fasting venous blood sample will be collected from each participant by a phlebotomist and analysed for Hb concentration, serum ferritin and C-reactive protein.

##### Determination of haemoglobin status

Hb level of participants will be measured using URIT 12 Hb analyser^(65,66)^. The test strip will be inserted into the URIT 12 Hb analyser and a drop of the drawn whole blood placed on the sample spot of test strip. The Hb results will be recorded to the nearest 0·1 g/dl. For girls aged ≥ 12 years, Hb levels < 8 g/dl, 8–10·99 g/dl, 11–11·9 g/dl and ≥ 12 g/dl will be categorised as severe anaemia, moderate anaemia, mild anaemia and normal, respectively^(67)^. For girls aged < 12 years, Hb levels < 8 g/dl, 8–10·9 g/dl, 11–11·4 g/dl and ≥ 11·5 g/dl will be categorised as severe anaemia, moderate anaemia, mild anaemia and normal, respectively^(68)^.

##### Determination of serum ferritin and C-reactive protein

The whole blood will be put in the gel-coated serum separator vacutainers and preserved at 4°C in an ice chest containing cold packs while in the field. The serum will be separated by means of a centrifuge at 500 rpm for 5 min at refrigerated temperature. The separated serum will be aliquoted and frozen at −80°C. The serum will be transported to the testing laboratory on frozen ice packs in an ice chest, when time is due for analysis. The ELISA technique will be used to assess serum concentrations of ferritin and C-reactive proteins. Fe deficiency will be defined as serum ferritin concentrations < 15 ug/l for healthy individuals and < 70 ug/l for individuals with inflammation/ infections^(55)^ and inflammation/infection as C-reactive proteins > 5 mg/l^(69)^.

#### Deworming

All participants will be treated against intestinal parasites by the study physician using a single dose of albendazole 400 mg chewable tablets. The first dose will be given 2 weeks prior to the commencement of feeding and a second dose given at midpoint of the intervention^(70)^.

#### Malaria screening and treatment

All participants will be tested for malaria using the malaria rapid diagnostic kits at baseline and halfway. Participants who test positive for malaria will be treated by the study physician using artemether-lumefantrine 20 mg/120 mg in 6-dose regimen over a 3-day period as recommended by Ghana Health Services^(71)^.

### Intervention

The beverage powders (fortified and unfortified) will be packaged in sealed plastic bags. Each participant in the intervention group will receive 1 pack of the baobab pulp-fortified pearl millet powder (60 g millet powder enriched with 9 g baobab pulp) daily. Similarly, each participant in the control group 1 will receive 1 pack of the unfortified pearl millet beverage powder (60 g millet powder) daily. Participants will prepare the beverage themselves by simply dissolving the powders in 300 ml of cold/chill water (sachet-treated water) and drink instantly in the presence of trained teachers and/or research assistants. As such, each participant will be provided with a cup, teaspoon and 300 ml cold/chill water for the preparation of the beverage. Participants will be fed the beverages once a day, five times a week (Monday to Friday) for six consecutive months (including school vacation time)^(38,52)^. Feeding will be done during the first break (10.00–10.30) and participants will be asked to report to the study team as soon as possible if they commence the intake of any Fe and folic acid supplements during the study period. Any leftover from each participant’s cup will be weighed using an electronic kitchen scale and the amount of beverage consumed computed as weight of beverage served minus weight of leftover.

In control group 2, participants will receive neither the baobab pulp-fortified pearl millet beverage nor the unfortified pearl millet beverage, but their usual Fe -folic acid tablets given by Ghana Health Service under the Girls’ Iron-Folate Tablet Supplementation Programme for the same duration, to allow for the efficacy of the fortified beverage to be evaluated in comparison with the standard Fe-folic acid supplementation regimen, as both treatments will provide roughly the same amount of Fe weekly. Hence, each participant in this group will receive 1 Fe-folic acid tablet (60 mgFe and 400 μg folic acid) once a week for six months. The girls will be given the tablets every Wednesday of the week after a lunch meal in the presence of teachers and/or research assistants^(72)^.

During school vacation time, the participants and their teacher will have to agree on a convenient time to come to school for the intake of the beverages or tablets^(38)^. For participants who fail to turn up during a feeding session, the teacher and/or field assistant will visit them to administer the beverage or tablets^(38)^.

Regarding criteria for discontinuing or modifying allocated interventions, the interventions will not be allowed to be modified and participants are required to consume all beverages/tablets provided in approved quantities, and can voluntarily withdraw at any time. Participants can also be permanently excluded for reasons such as refusal to partake in any required study procedures.

Also, all participants will be sensitised on the benefits of the interventions, as a crucial first step to improving compliance and continued participation (participant retention). The study team will also be available throughout the trial period to identify and address any barriers to compliance and continued participation. Participants will also be made to consume the beverages/tablets in the presence of the study team to ensure compliance.

### Mid-way treatment

All participants will also be re-treated against intestinal parasites at mid-way using a single dose of albendazole 400 mg chewable tablets. Participants will also be re-tested for malaria at midpoint. Those who test positive for malaria will be treated by the study physician.

### Post-intervention assessment

At the end of the feeding intervention, post-intervention assessment will be done immediately. Dietary intake, anthropometry and the biochemical indices will be reassessed.

### Outcome measures

The primary outcomes will be serum ferritin (ug/l) and Hb (g/dl) concentrations of participants. Secondary outcomes will be prevalence of Fe deficiency, Fe deficiency anaemia and BMI-for-age.

### Statistical analysis

Data analysis will be done using Statistical Package for Social Sciences version 22 and WHO AnthroPlus software. Shapiro–Wilk and Kolmogorov–Smirnov tests will be done to test quantitative data for normality. Data that turns out not to be normally distributed will be analysed using non-parametric tests. Continuous data will be reported as means and sd, whilst categorical variables will be presented as frequencies and percentages. Data will be analysed using intention to treat analysis. One-way ANOVA will be used to compare means of serum ferritin and Hb levels among the three study groups (arms). Tukey’s post hoc test of one-way ANOVA will be performed to determine intergroup differences. Paired *t* test will be used to determine change in means of serum ferritin and Hb levels within groups (baseline and end-line). Relationship between covariates and outcome variables will be determined using multivariate linear and binary logistic regression analyses. WHO AnthroPlus software will be used to compute BMI-for-age Z-scores to determine participant’s nutritional status. Missing data will be resolved via multiple imputations. *P* < 0·05 will be considered significant at two-tailed tests.

### Data monitoring

The dietary intervention, which participants are accustomed to, and the other assessments pose minimal risk to participants. As such, a steering committee will not be formed, since no severe adverse events are anticipated. The principal investigator, the study physician and the other study investigators will regularly monitor for adverse effects and progress of the trial.

### Adverse events

Though the risks of partaking in this feeding trial are low, all participants will be monitored and any suspected adverse events will be attended to by the study physician. The study physician will assess participants every week for any adverse events. Participants will also be provided with the contact information of the lead investigator and study physician to allow for rapid reporting of adverse reactions. All adverse events will be documented in the daily case report form and reported regularly to the ethics committee.

### Ethics and dissemination

This study will be conducted according to the guidelines laid down in the Declaration of Helsinki, and all procedures involving human subjects/patients were approved by the Committee on Human Research, Publications and Ethics (CHRPE/AP/279/23) at Kwame Nkrumah University of Science and Technology, Ghana. Written consent of participants and their guardians will be obtained by research assistants. Prior to the consent taking, the researchers will provide the participants and their guardians with enough information about the study including risks and benefits and assure them of confidentiality. Partaking in the study will be made voluntary and respondents can choose to withdraw from the research at any time. The trial is registered with the Pan African Clinical Trial Registry (https://pactr.samrc.ac.za/), with registration number PACTR202306743290094 and a version date of February 16, 2024. The protocol is written based on Standard Protocol Items: Recommendations for Interventional Trials guidelines^(73)^. To maintain confidentiality, code numbers will be assigned to all participants; hence, no name will be recorded. Data collected will be stored appropriately, locked and accessible to only the researchers. Any changes to the protocol will be communicated to the ethics committee and funder for approval prior to implementation. Study findings will be disseminated through peer-reviewed publications, with copies placed in Kwame Nkrumah University of Science and Technology’s libraries. Additionally, copies will also be placed at the Kumbungu District Education Office and Kumbungu District Health Directorate. The principal investigator will manage authorship based on the study team’s tasks and there is no intention to use professional writers. The trial data and informed consent materials will be made available on reasonable request from the principal investigator; however, personally identifiable information will not be released.

## Discussion

This study aims to investigate the efficacy of baobab pulp-fortified pearl millet beverage powder in improving Fe and anaemia status of adolescent girls. Anaemia is a significant health concern among women worldwide, especially among adolescent girls,^(7)^ and greatly affects their health outcomes^(6)^. Adolescent girls’ rapid physical and cognitive growth and work performance require high oxygen-dependent energy supply and metabolism^(74)^; however, anaemia decreases oxygen-dependent cellular energy metabolism, resulting in impaired linear growth, reduced cognitive abilities and poor work performance^(74)^, all of which can impede a nation’s overall economic productivity both now and in the future^(6)^. Adolescent girls with Fe deficiency anaemia are also at higher risk of infection due to impaired immune system function, as Fe is required for the regulation of macrophage polarisation, neutrophils recruitment, natural killer cell activity, B-cells antibody response and T-cell activation^(75,76)^. Moreover, when anaemic adolescent girls get pregnant, they are exposed to a higher risk of maternal morbidity and mortality and adverse pregnancy outcomes, including preterm birth, low birth weight and perinatal death^(77)^. Thus, if this study establishes therapeutic efficacy, it will contribute to the reduction of anaemia and Fe deficiency in teenage girls, improving their health outcomes.

The main conventional methods for addressing Fe deficiency anaemia among adolescent girls such as Fe food fortification and Fe supplementation are known to be less acceptable and sustainable in developing countries as compared with food-to-food fortification, as food-to-food fortification utilises locally available foods^(16,17)^. The food vehicle (pearl millet) and fortificant (baobab fruit pulp) of this study are readily available year-round in Ghana, as they are grown locally as food. Thus, the utilisation of these locally available foods may make the intervention culturally acceptable and sustainable years after it ends. Hence, this study may identify an acceptable, sustainable and cost-effective way of reducing or preventing Fe deficiency anaemia among adolescent girls.

Monotonous plant-based diets, especially unrefined cereal-based diets, which are widely consumed in many low-income countries^(78)^, have low bioavailable Fe due to elevated levels of Fe absorption inhibitors, particularly phytate^(79,80)^. Phytate chelates Fe in the digestive tract, making it less bioavailable for absorption^(80)^. Consuming Fe-rich plant-based foods together with vitamin C and citric acid-rich foods is therefore recommended to improve Fe absorption^(81)^, as vitamin C and citric acid mitigate the formation of insoluble Fe complexes and reduce ferric to ferrous Fe, which is essential for Fe absorption^(26,27)^. Although it has been shown that vitamin C and citric acid-rich baobab fruit pulp enhance Fe bioavailability when combined with Fe-rich pearl millet flour^(28,29)^, these findings have only been shown *in vitro*. As such, this will be the first study in humans to test the effect of baobab pulp-fortified pearl millet beverage on Fe and anaemia status. The demand for a more widely accepted and long-lasting intervention to address anaemia and Fe deficiency is growing, making the identification of a food-to-food intervention crucial.

The study has potential limitations worth mentioning. The study design that allows participants, investigators, research assistants and teachers to be unblinded may introduce potential bias into the study. Participants’ awareness of group assignment can influence their behaviour in a way that can affect the research outcome^(82)^. Also, if the individuals giving the treatment are aware of the group assignment, they might treat respondents differently, influencing the final outcomes either directly or indirectly^(82)^. To minimise the risk of bias, participants will be informed that all three treatments contain critical nutrients required for adolescent health and as well encourage them to take their assigned supplements regularly^(83)^. The study team will also be urged to give equal support and encouragement to participants in all the arms of the trial^(83)^. Also, as the dietary intake assessment methods of the study are reliant on memory, there is a risk of recall bias. However, with the help of the participants’ mothers, who will be invited to be present during dietary data collection, the risk of recall bias will be minimised. Also, given that dietary intake is to be self-reported, response bias may affect the exact estimation of food intake. To minimise the risk of self-reported bias, interviewers will be thoroughly trained in rapport-building. Effective rapport-building will enable participants to feel more comfortable opening up and responding truthfully to questions^(84)^.

## Supporting information

Atosona et al. supplementary material 1 Atosona et al. supplementary material

Atosona et al. supplementary material 2 Atosona et al. supplementary material

Atosona et al. supplementary material 3 Atosona et al. supplementary material

## Supplementary material

For supplementary material/s referred to in this article, please visit https://doi.org/10.1017/S0007114524001430
