# Supplementary material for: Pearl millet instant beverage powder enriched with baobab pulp to improve iron and anaemia status of adolescent girls in rural Ghana: a study protocol for a cluster randomised controlled trial
Source: Br J Nutr. 2024 Sep 19;132(5):565–74. doi: 10.1017/S0007114524001430 (PMC11531939; doi:10.1017/S0007114524001430)
Supplement: Atosona et al. supplementary material 1 — Atosona et al. supplementary material [file S0007114524001430sup001.docx]

**Supplementary file 1: Sensory attributes scores for beverage samples enriched with 0, 10, 15 and 20g of baobab powder**

| **Attribute** | **S1(0g)** | **S2 (10g)** | **S3 (15g)** | **S4 (20g)** | **P-value** |
| --- | --- | --- | --- | --- | --- |
| **Taste/sweetness** | 7.3±1.2 | 7.5±1.1 | 7.8±1.1 | 7.9±1.5 | 0.099 |
| **Colour** | 7.8±1.1 | 7.6±1.2 | 7.9±1.4 | 7.6±1.7 | 0.722 |
| **Flavour** | 7.8±1.1 | 7.6±1.4 | 7.8±1.2 | 7.6±1.6 | 0.866 |
| **Sourness** | 8.0±1.1 | 7.8±1.1 | 7.6±1.3 | 6.4±2.4 | <0.001 |
| **General acceptability** | 7.7±1.0 | 7.8±1.2 | 8.1±1.0 | 6.9±1.7 | <0.001 |
