# Supplementary material for: Pearl millet instant beverage powder enriched with baobab pulp to improve iron and anaemia status of adolescent girls in rural Ghana: a study protocol for a cluster randomised controlled trial
Source: Br J Nutr. 2024 Sep 19;132(5):565–74. doi: 10.1017/S0007114524001430 (PMC11531939; doi:10.1017/S0007114524001430)
Supplement: Atosona et al. supplementary material 2 — Atosona et al. supplementary material [file S0007114524001430sup002.docx]

**Supplementary file 2: Microbial quality of baobab pulp-fortified pearl millet powder**

| **Test** | **Results (CFU/g)** | **Safe limit(CFU/g)** |
| --- | --- | --- |
| Total aerobic count | 1.50×${10}^{3}$ ± 0.04 | ≤1.00 ×${10}^{6}$ |
| Total coliform count | 0.00 | - |
| Yeasts and mold count | 1.50×${10}^{2}$ ± 0.00 | ≤1.00x${10}^{4}$ |
| Staphylococcal count | 1.00×${10}^{1}$ ± 0.00 | ≤1.00x${10}^{3}$ |
