# Supplementary material for: Pearl millet instant beverage powder enriched with baobab pulp to improve iron and anaemia status of adolescent girls in rural Ghana: a study protocol for a cluster randomised controlled trial
Source: Br J Nutr. 2024 Sep 19;132(5):565–74. doi: 10.1017/S0007114524001430 (PMC11531939; doi:10.1017/S0007114524001430)
Supplement: Atosona et al. supplementary material 3 — Atosona et al. supplementary material [file S0007114524001430sup003.pdf]

KWAME NKRUMAH UNIVERSITY OF SCIENCE AND TECHNOLOGY
COLLEGE OF SCIENCE
DEPARTMENT OF BIOCHEMISTRY AND BIOTECHNOLOGY

A RESEARCH QUESTIONNAIRE

Dietary intake, knowledge and practices on iron deficiency anaemia and anthropometry of adolescent girls in rural Ghana

---

Introduction
I ...., wish to have a conversation with you on the above topic. Please be candid and truthful in your response and be assured that what is being discussed will remain confidential and shall be used for the purpose of this research only.
Thank you.

Section A: Identification

1. Respondent ID: ……………………….
2. Date of interview (dd/mm/yy): ….. /….. /….
3. School code: ………..
4. Interviewer code: ………
5. Questionnaire code: ………

Section B: General characteristics of respondent

6. What is your age (years) …………………...

7. What is your level of education?
   a. Primary [ ]
   b. JHS [ ]

5. Which religion do you belong?
   a. Christianity
   b. Islam
   c. ATR
   d. Others (specify): _____________

6. What is your ethnicity?
   a. Dagomba
   b. Gonja
   c. Mamprusi
   d. Others, specify: ___________________

7. Who do you live with? If mother, skip to question 10, if guardian, skip to question 12.
   a. Both parents
   b. Father
   c. Mother

d. Guardian (specify) ……………

8. What is your father's highest educational level completed?
    a. None
    b. Primary
    c. Middle/ J.H.S
    d. S.H.S/Vocational training
    e. Tertiary
    f. Others (specify) ______________________

9. What is your father's current occupation?
    a. Not currently working
    b. Farmer
    c. Salary/office worker (professional)
    d. Trader
    e. Self-employed (e.g. carpenter, mason, seamstress, tailor and any other artisans)
    f. Other (Specify) …………………

10. What is your mother's highest educational level completed?
    a. None
    b. Primary
    c. Middle/ J.H.S
    d. S.H.S/Vocational training
    e. Tertiary
    f. Others (specify) ______________________

11. What is your mother's current occupation?
    a. Not currently working
    b. Farmer
    c. Salary/office worker (professional)
    d. Trader
    e. Self-employed (e.g. carpenter, mason, seamstress, tailor and any other artisans)
    f. Other (Specify) …………………

12. What is your guardian's highest educational level completed?
    a. None
    b. Primary
    c. Middle/ J.H.S
    d. S.H.S/Vocational training
    e. Tertiary
    f. Others (specify) ______________________

13. What is your guardian's current occupation?
    g. Not currently working

h. Farmer
i. Salary/office worker (professional)
j. Trader
k. Self-employed (e.g. carpenter, mason, seamstress, tailor and any other artisans)
l. Other (Specify) …………………

14. What is the size of your household?
    a. <5
    b. >5
15. Have you started menstruating?
    a. Yes
    b. No
16. If yes, how old were you when you had your first menstruation? …………………

SECTION C: DIETARY ASSESSMENT

24 hour dietary recall

17a. Please describe all foods that you ate yesterday (past 24 hours beginning from the moment you woke up till you went to bed) at home and outside of the home. Please start with the first food eaten in the morning.

**Eating moment**	**Name of dish**	**Quantity of dish consumed (Household measurement)**	**Ingredients used**
Breakfast			
Snack before lunch			
Lunch			
Snack before dinner			

Dinner			
Snack after dinner			
Drinks			

17b. Based on the meals recorded above (17a), indicate whether the respondent ate from the following food groups during the past 24 hours.

**Q. No**	**Food group**	**Examples**	**Yes=1 No=0**
**1**	**Grains, white roots and tubers, and plantains**	*Guinea corn, corn/maize, rice, wheat, sorghum, millet, plantain, yam, cassava, white sweet potato, or any other grains or foods made from these (e.g. bread, noodles, porridge, TZ, banku, kenkey, fufu, gari or other grain/root tuber products)*	
**2**	**Pulses (beans, peas and lentils)**	*beans, peas, lentils, cowpea, soybean, bambara beans, pigeon peas,*	
**3**	**Nuts and seeds**	*nuts, seeds, groundnuts, cashew nut, Bungu (sesami), neri (melon seeds) or foods made from these*	
**4**	**Dairy**	*Milk, powdered milk, cheese, yogurt or other milk products*	
**5**	**Meat, poultry and fish**	*Beef, lamb, goat, rabbit, wild game, chicken, duck, guinea fowl or other birds, fresh or dried fish or shellfish, yurayura (keta school boys), any organ meat (e.g. liver, kidney, intestines, heart or other organ meats or blood-based foods)*	
**6**	**Eggs**	*Chicken, duck, guinea fowl or any other egg*	
**7**	**Dark green leafy vegetables**	*dark green/leafy vegetables, including wild ones + locally available vitamin-A rich leaves such as bra, amaranth, cassava leaves, cowpea leaves, onion leaf, ayoyo, alefu, moringa leaves, bitter leaves, baobab*	

		leaves (fresh and/or dry), pumpkin leaves and fruit, okra leaves and any other fresh dark green leafy vegetables.	
**8**	**Other vitamin A-rich fruits and vegetables**	*Tomato, carrots, or sweet potatoes that are orange inside + other locally available vitamin-A rich vegetables (e.g. red sweet pepper), and unripe/green beans, ripe mangoes, ripe papaya, sheanut fruits, watermelon, dawadawa pulp and other locally available vitamin A-rich fruits,*	
**9**	**Other vegetables**	*other vegetables including wild vegetables, okra/okro fruit (fresh and/or dry), onion, garden eggs, dry baobab leaves (kuka), lettuce, green pepper, red pepper, garlic*	
**10**	**Other fruits**	*Other fruits including wild fruits pineapple, apple, cashew fruits, ebony fruits,*	
**Total dietary diversity score (DDS)**			

Dietary habits

18. Interviewer: Please write down the most appropriate answer in the box corresponding to question for weekdays and weekends

		**Week days (Monday-Friday)**<br>0=Never<br>1=1 day<br>2=2 days<br>3=3 days<br>4=4 days<br>5=5 days	**Weekend (Saturday and Sunday)**<br>0=Never<br>1=Saturday<br>2=Sunday<br>3=Both Saturdays and Sundays
1	How often do you usually have breakfast?		
2	How often do you usually have lunch?		
3	How often do you eat supper/dinner?		

Dietary pattern

19. I would like to know how often you eat the following types of food

**Food**		**Frequency of consumption (Interviewer, tick where appropriate)**					
		Never	Once per month	Once per week	2-4 days/week	5-6 days/week	Daily
1	Fruits						
2	Vegetables						

3	Sweets (e.g. candies or coffees)						
4	Sugar-sweetened drinks/beverages (e.g. coke, fanta, kalypo etc.)						
5	Savoury and fried snacks (e.g. doughnuts, pastries, pie etc.)						
6	Dairy products						
7	Meat						
8	Eggs						
9	Fish						
10	Pulses (beans, peas and lentils) and nuts/seeds						
11	Cereals, grains and tubers						

SECTION D: KNOWLEDGE ON IRON DEFICIENCY ANAEMIA

Please I am going to ask you some questions about anaemia and iron-rich foods. Please let me know if you need me to clarify any of my questions. Feel free to ask any question you may have.

General signs of iron-deficiency anaemia

20. Have you heard about iron-deficiency anaemia? If ‘no’/’don’t know’ skip to question 29
    1. Yes 2. No 3. Don’t know/no answer

21. If Yes: Can you tell me how you can recognize someone who has anaemia?
    1. Less energy/weakness
    2. Spoon nails/bent nails (koilonychia)
    3. Paleness/pallor
    4. More likely to become sick (less immunity to infections)
    5. Other 6. Don’t know

Causes of iron-deficiency anaemia (tick as many as you know)

22. What causes anaemia?
    a. Lack of iron in the diet/eat too little, not much
    b. Sickness/infection (malaria, hookworm infection, other infection such as HIV/AIDS)
    c. Heavy bleeding during menstruation
    d. Other
    e. Don’t know

Consequences of iron-deficiency anaemia for young children and pregnant women

23. What are the health risks for young children of a lack of iron in the diet?
   a. Delay of mental and physical development
   b. Other
   c. Don’t know

24. What are the health risks for pregnant women of a lack of iron in the diet?
   a. Risk of dying during or after pregnancy
   b. Difficult delivery
   c. Other
   d. Don’t know

Prevention of anaemia

25. How can anaemia be prevented?

   a. Eat/feed iron-rich foods/having a diet rich in iron
   b. Eat/give vitamin-C-rich foods during or right after meals
   c. Take/give iron supplements if prescribed
   d. Treat other causes of anaemia (diseases and infections) – seek health-care assistance
   e. Other
   f. Don’t know

Iron-rich foods – easily absorbed (tick as many as you know)

26. Can you list examples of foods rich in iron? ....

Organ meat
   a. Liver
   b. Kidney
   c. Heart

Flesh meat
   a. Beef
   b. Pork
   c. Lamb
   d. Goat
   e. Rabbit
   f. Dog
   g. Chicken
   h. Duck

Insects
   a. Insect larvae
   b. Red ants
   c. Grasshoppers
   d. Crickets
   e. Termite

Fish and seafood
   a. Fresh fish
   b. Dried fish
   c. Canned fish
   d. Prawns
   e. Shrimps

f. Seafood

Foods that increase iron absorption

27. When taken during meals, certain foods help the body absorb and use iron. What are those foods?

a. Vitamin-C-rich foods, such as fresh citrus fruits (orange, lemons, tangerine, grapefruit etc.).
b. Other
c. Don’t know

Foods that decrease iron absorption

28. Some beverages decrease iron absorption when taken with meals. Which ones?

a. Coffee
b. Tea
c. Other
d. Don’t know

SECTION E: PREVENTION PRACTICES RELATED TO IRON DEFICIENCY ANAEMIA

Food-intake practices

I would like to ask you about particular foods you may eat on your own or as part of a dish.

29. Yesterday, during the day and night, did you eat any of the following? (Read the list of iron-rich foods and circle either yes or no for each food item).

Organ meat

Liver	a. Yes	b. No
Kidney	a. Yes	b. No
Heart	a. Yes	b. No

Flesh meat

Beef	a. Yes	b. No
Pork	a. Yes	b. No
Lamb	a. Yes	b. No
Goat	a. Yes	b. No
Rabbit	a. Yes	b. No
Dog	a. Yes	b. No
Chicken	a. Yes	b. No
Duck	a. Yes	b. No

Insects

Insect larvae	a. Yes	b. No
Red ants	a. Yes	b. No

Grasshoppers	a. Yes	b. No
Crickets	a. Yes	b. No
Termites	a. Yes	b. No

Fish and seafood

Fresh fish	a. Yes	b. No
Dried fish	a. Yes	b. No
Canned fish	a. Yes	b. No
Prawns	a. Yes	b. No
Shrimps	a. Yes	b. No
Seafood	a. Yes	b. No

Consumption of vitamin-C-rich fruits

30. Do you usually eat fresh citrus fruits, such as oranges, tangerine, lemon, grapefruit etc, or drink juice made from them? If no or don't know/no answer, skip to question 25.

a. Yes
a. No
b. Don't know/no answer

31. If Yes: Every day?

a. Yes
b. No
c. Don't know/no answer

32. When do you usually eat fresh citrus fruits? (Read the following options to the respondent)

a. Before a meal
b. During the meal
c. After a meal
d. Other (specify) ________________________________
e. Don't know/no answer

Consumption of coffee/tea

33. Do you usually drink coffee or tea? If 'no' or 'don't know' skip to section F.

a. Yes
b. No
c. Don't know

34. If Yes, every day?

a. Yes
b. No
c. Don't know

35. When do you usually drink coffee or tea? (Read the following options to the respondent)

a. Two hours or more before a meal
b. Right before a meal
c. During the meal

d. Right after a meal
e. Two hours or more after a meal
f. Other (specify) ________________________
g. Don't know/no answer

SECTION F: ANTHROPOMETRY OF RESPONDENT

36. Height (m): ……………
37. Weight (kg): ………….
38. BMI (kg/m2): …………
